# Physiological responses induced by phospholipase C isoform 5 upon heat stress in *Arabidopsis thaliana*

**DOI:** 10.3389/fpls.2023.1076331

**Published:** 2023-01-25

**Authors:** Nazish Annum, Moddassir Ahmed, Mark Tester, Zahid Mukhtar, Nasir Ahmad Saeed

**Affiliations:** ^1^ Wheat Biotechnology Lab, Agricultural Biotechnology Division, National Institute for Biotechnology and Genetic Engineering Constituent College (NIBGE-C), Pakistan Institute of Engineering and Applied Sciences (PIEAS), Faisalabad, Pakistan; ^2^ Center for Desert Agriculture (CDA), King Abdullah University of Science and Technology (KAUST), Thuwal, Saudi Arabia

**Keywords:** PG, PIP_2_, Thermotolerance, *Arabidopsis thaliana*, Root growth

## Abstract

Plant’s perception of heat stress involves several pathways and signaling molecules, such as phosphoinositide, which is derived from structural membrane lipids phosphatidylinositol. Phospholipase C (PLC) is a well-known signaling enzyme containing many isoforms in different organisms. In the present study, Phospholipase C Isoform 5 (*PLC5*) was investigated for its role in thermotolerance in *Arabidopsis thaliana*. Two over-expressing lines and one knock-down mutant of *PLC5* were first treated at a moderate temperature (37 °C) and left for recovery. Then again exposed to a high temperature (45 °C) to check the seedling viability and chlorophyll contents. Root behavior and changes in ^32^P_i_ labeled phospholipids were investigated after their exposure to high temperatures. Over-expression of *PLC5* (*PLC5 OE*) exhibited quick and better phenotypic recovery with bigger and greener leaves followed by chlorophyll contents as compared to wild-type (*Col-0*) and *PLC5* knock-down mutant in which seedling recovery was compromised. *PLC5* knock-down mutant illustrated well-developed root architecture under controlled conditions but stunted secondary roots under heat stress as compared to over-expressing *PLC5* lines. Around 2.3-fold increase in phosphatidylinositol 4,5-bisphosphate level was observed in *PLC5 OE* lines upon heat stress compared to wild-type and *PLC5* knock-down mutant lines. A significant increase in phosphatidylglycerol was also observed in *PLC5 OE* lines as compared to *Col-0* and *PLC5* knock-down mutant lines. The results of the present study demonstrated that *PLC5* over-expression contributes to heat stress tolerance while maintaining its photosynthetic activity and is also observed to be associated with primary and secondary root growth in *Arabidopsis thaliana*.

## 1 Introduction

Temperature plays an important role in plant growth and productivity. Atmospheric temperature of earth is continually changing round the year and it is becoming quite distinct in the event of climate change. The frequency and magnitude of heat-wave events in the past decades point toward an alarming increase in the global mean temperature (3.7 ± 1.1°C) by the end of the 21^st^ century (Zhu et al., 2022). So, it has become increasingly important to understand how plants respond to high temperatures. Usually, high temperature affects a variety of potential cellular targets, distressing plant growth and survival. In a normal atmosphere, plants experience day-to-day and periodic temperature changes that vary in range, frequency and magnitude. *Arabidopsis thaliana* as a model plant has differences in sensitivity and response to temperature extremities that help the plant adapt to changing local temperature patterns. Gene expression changes under rising temperature, leading to high-temperature tolerance. The genetic reprogramming and successive increase in thermotolerance are known as adaptive and/or acquired thermotolerance (Zhang et al., 2015). At the cellular level, high temperature leads to protein misfolding, perturbs membrane fluidity, transport and enzymatic reaction, cytoskeleton organization and metabolic balance by accumulating reactive oxygen species (ROS). Thus, as sessile eukaryotes, plants quickly sense temperature fluctuations in the environment and recruit timely adaptive tactics to preserve cell function and viability (Hayes et al., 2021).

Membrane plays an important role in vesicle transport and cell signaling, not only through host-specific proteins but also provides a substrate for the production of lipids (as a second messenger). Besides playing structural role as membrane components, lipids perform regulatory and signaling functions, thus activating cellular responses to environmental signals (Hou et al., 2016; Kosová et al., 2018). PIP_2_ (Phosphatidylinositol 4,5-bisphosphate) and PA (Phosphatidic acid) lipids and their associated metabolic enzymes like PLC, PLD, DGK (Diacylglycerol Kinase) and PIP5K (Phosphatidylinositol 4 phosphate 5 kinase) have various regulatory and cellular functions in response to environmental stimuli. However, plants PLC signaling system are presumed to be different from animals in terms of lacking the primary targets for DAG and IP3, i.e. PKCs, TRP channels and IP3 receptors (Wheeler and Brownlee, 2008; Munnik, 2014). PIP_2_ has been hardly detected in the plasma membrane of flowering plants which is supposed to be the substrate of PLC. PIP is also assumed to be a precursor of PLC as it is abundantly observed in the plasma membrane. Yet it is still debatable that, what is the typical precursor of PLC (Munnik et al., 1998; Munnik, 2014; Simon et al., 2014).

Acting very early in the response pathway, PLCs assumed to be physically close to the thermosensor. Phospholipases use PIP_2_/PIP as a substrate to generate DAG and IP_s_ (Inositol phosphates) that could ultimately help in the activation of calcium (Ca^+2^) channels, which in turn activates various sHSP (small heat shock proteins) and HSR (heat shock responsive) genes through various pathways. Furthermore, CaBP (calcium binding proteins), also known as “calcium sensor” recognize and decrypt the existing information in the calcium signatures. This information is then transported to initiate downstream phosphorylation cascade in order to regulate gene expression (Tuteja and Sopory, 2008; Niu and Xiang, 2018). Previously, (Hunt et al., 2004) reported the differential calcium sensitivities for AtPLC protein activities which may act as mechanistic attributes for generated calcium signatures. Beside PIP_2_/PIP, PI is also a component of signaling system and take part in signal perception and transduction (Liu et al., 2019). Phosphatidylcholine (PC) and phosphatidyl ethanol (PE) ratio affects membrane stability (Yu et al., 2020). Phosphatidylglycerol (PG) was reported to be an important and major lipid component of chloroplast membrane (Demé et al., 2014). The level of PG was reported to be decreased under heat treatment to maintain chloroplast membrane stability and high photosynthetic activity (Zhu et al., 2022). Similarly, cardiolipin (CL) involved in various mitochondrial events, is also known as the signature phospholipid of mitochondria (Mileykovskaya and Dowhan, 2014).

Plant PLC signals in response to various abiotic stresses including cold, drought, and salt (Darwish et al., 2009; Arisz et al., 2013; Simon et al., 2014) to elicit various downstream mechanism to cope with these environmental stresses. Nine PLCs were reported to be found in *Arabidopsis thaliana*. Each PLC has unique role and tested against various stresses. Previously, *PLC2* has been reported to be involved in ER stress response pathway (Kanehara et al., 2015). Similarly, PLC involvement in thermotolerance had also been reported, where knockout mutant of *PLC3* and *PLC9* exhibited severely impaired basal and acquired thermotolerance, while their over-expression improved thermotolerance (Zheng et al., 2012; Gao et al., 2014; Ren et al., 2017). *PLC3* also reported to be involved in ABA (abscisic acid) signaling (Zhang et al., 2018a), likewise *PLC4* found to be a negative regulator of salt tolerance (Xia et al., 2017), while over-expression of *PLC5* improved drought tolerance (Zhang et al., 2018b). Although *AtPLC5* (multi stress tolerant gene) expression is reported to be induced by dehydration, salt and cold stresses (Hunt et al., 2004), it is yet to be tested against thermotolerance. The current study deals with evaluation of thermotolerance in *PLC5* mutant plants at seedling stage. The over expressed *PLC5* lines demonstrated improved plant vigor and enhanced recovery when subjected to acquired thermotolerance. In addition, *PLC5 OE* lines exhibited improved primary and lateral root growth (**Supplementary data**) as compared to knock down mutants and wild type plants when exposed to heat shock. This could be associated to less damage of chloroplast and thylakoid membranes and thus to less disruption in light capturing and photosynthetic activity as it has been described (Kato et al., 2020). In our case, this was supported by a decrease of chlorophyll amount in the wild type and Knocked-down mutant (which are sensitive to the heat treatments).

## 2 Materials and methods

### 2.1 Plant material

The seeds of *Arabidopsis thaliana* (*Col-0*) and its homozygous *AtPLC5* over-expressing and knockdown mutants *i.e. OE_2_*, *OE_3_* and *K_d_* were kindly provided by Dr. Teun Munnik, Research Cluster Green Life Sciences, Section Plant Cell Biology, Swammerdam Institute for Life Sciences, University of Amsterdam, Netherlands.

### 2.2 Seed sterilization

The seeds of *Arabidopsis thaliana* and its *AtPLC5* mutants were surface sterilized in a desiccator by using commercial bleach and 37% HCl for 3 h and then placed in a laminar air flow hood for an hour to allow complete evaporation of chlorine gas. This method is also called the “Dry method of seed sterilization” or “Chlorine gas seed sterilization” (Zhang et al., 2018b).

### 2.3 Seedling survival assay for acquired thermotolerance

Sterilized seeds were placed in round petri plates containing ½ strength MS medium (Murashige and Skoog, 1962) including Gamborg B_5_Vitamins and 1% sucrose. Stratification was done at 4 °C in dark for 2 days. After stratification, petri plates containing seeds were placed at 22 °C (16/8 h light-dark period) in a growth chamber for 3 days to grow and then subjected to heat shock. Seedlings were first treated at 37 °C for 60 min, then placed at 22 °C for 120 min for recovery, and then again subjected to a new treatment at 45 °C for 0 min (Control) and 150 min (Heat shock). A water bath was used for heat shock treatment. After heat treatment, seeds were put at 22 °C (16/8 h light-dark period) to recover for 10-14 days. The seedling survival rate was calculated based on the visual color of tissue, as green seedlings were considered as alive and white seedlings as dead.

### 2.4 Determination of chlorophyll levels

The aerial part of the fresh seedling was collected in a 2 ml Eppendorf tube and ground partially with the help of a small pestle in order to facilitate the carotenoid extraction. Acetone (80%) was used for chlorophyll extraction and kept under dark conditions for 1.45 h in order to ensure maximum pigment diffusion. After centrifugation (12000xg for 2 min) the supernatant was collected and absorbance (A_x_) was measured using a Spectrophotometer (U 5100 Spectrophotometer, Hitachi) at 663.2 nm and 646.8 nm wavelength by taking 80% acetone as a blank. Chlorophyll concentration was estimated by following (Lichtenthaler, 1987) protocol.

### 2.5 Root elongation assay

For root elongation assay Square Petri plates (12 x 12 cm) containing media described previously, were used. Seeds were put on Square Petri plates and stratified at 4 °C in a dark room for 2 days, then placed in a growth chamber vertically tilted at 70 degrees angle at 22 °C with 16/8 h light-dark period for 3 days before heat treatment. Sealed Square Petri plates were placed in a water bath for heat treatment at 44 °C for 0 min (Control) and for 36 min (Heat shock) in order to test the basal thermotolerance. Plates were scanned on daily basis before and after the heat treatment by using Epson Perfection V800 photo scanner, at 300 DPI for 12 days. *Smart Root* version 4.1 (Lobet et al., 2011) plugins of the *Fiji Image J* (RRID: SCR_003070, https://imagej.net/software/fiji/) software (Schindelin et al., 2012) was used to measure the root growth.

### 2.6 Radioactive phospholipid labeling, extraction and analysis

Five-day old seedlings of *Arabidopsis thaliana* (*Col-0*) and its *AtPLC5* mutants were metabolically labeled in 2 ml Eppendorf tubes by using 200 μl labeling buffer (MES-KOH 2.5 mM, pH 5.8, KCl 1 mM) containing carrier free ^32^PO_4_^3-^ (5-10 μCi) for overnight incubation. Three biological replicates were used per genotype for simple randomization. Three seedlings were used for each replicate. After overnight incubation for ^32^P_i_ labeling, samples were subjected to heat shock at 40 °C for 30 min using heating block. Perchloric acid (5% v/v) was then added to stop the treatment and lipids were extracted and separated by following protocol of (Munnik and Zarza, 2013). Heat activated potassium oxalate impregnated thin layer chromatographic (TLC) plates were used to separate the lipids (Munnik et al., 1998). Extracted lipids were visualized on autoradiograph by overnight exposure of TLC plate to autoradiography film and quantified by using phosphoimaging (Typhon FLA 7000, GE Healthcare). Individual phospholipid level was determined as the percent fraction of total lipids.

### 2.7 Statistical analysis

Statistical analysis was performed using Microsoft Excel 2016 for Window 10 (RRID: SCR_016137, https://www.microsoft.com/en-gb/). Significance of data was determined using one way ANOVA with student’s t test (***P <0.001).

## 3 Results

### 3.1 Seedling survival

Upon heat perception different downstream complex mechanisms are activated in plants, suggesting that various genes may be involved in thermotolerance. *Arabidopsis thaliana*, as a model system, is generally used for phenotypic characterization by different phenotypic assays (Silva-Correia et al., 2014). Among this seedling survival was one of the most regularly used method for evaluating heat tolerance in *Arabidopsis thaliana* (Yeh et al., 2012). Survival is usually scored based on the color of the tissue. Seedlings that remain green after being exposed to heat/high temperature stress and are actively growing and producing new leaves are considered viable. In contrast, yellowing or whitening of leaves demonstrate non-viable seedling because of their impaired photosynthetic activity (Zheng et al., 2012; Silva-Correia et al., 2014).

### 3.2 Acquired thermotolerance

**Figure 1:**
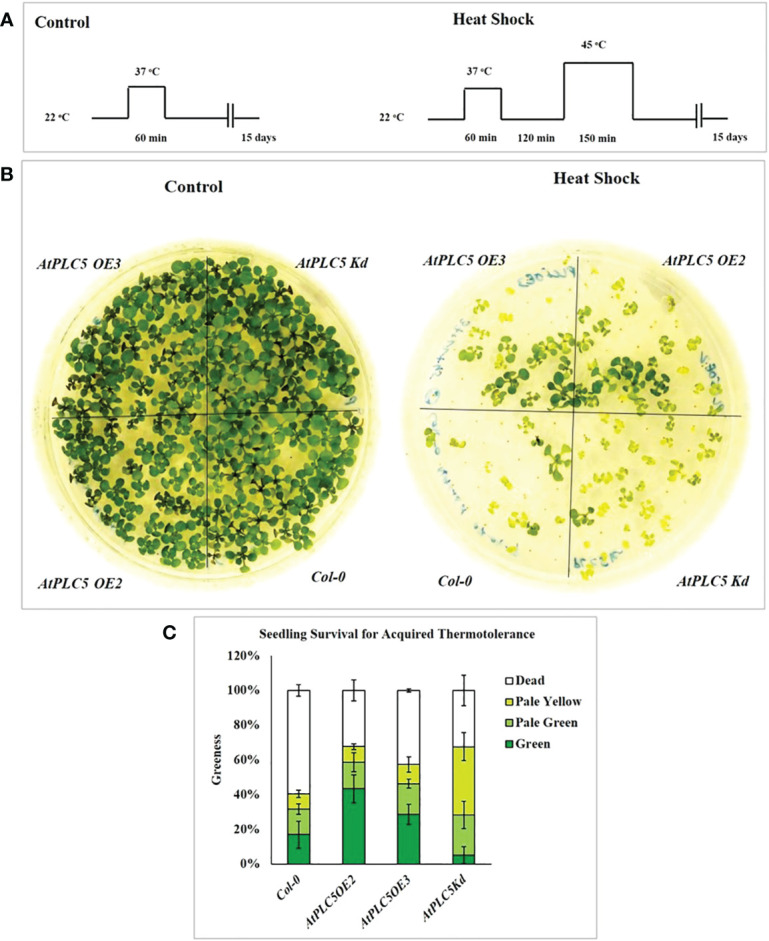
Seedling survival assay for acquired thermotolerance. **(A)** Schematic diagram of the heat shock conditions used to assess acquired thermotolerance phenotypes of *AtPLC5* mutants of *Arabidopsis thaliana.* Three days old seedlings were prior acclimatized by subjected to heat stress at 37 °C for 1 h, at 22 °C for 2 h (for recovery) and then exposed to 45 °C for 0 h (control) and 2.5 h (heat shock). Seedlings of mutants were daily monitored for 15 days after treatment. **(B)** Survival was observed and scored based on seedling leaves color. **(C)** Mean derived from 4 replicates, containing 34 seedlings for each genotype tested. Error bar represent standard deviation of the mean. Experiment was repeated 3 times independently. Data was represented in percentage.

In present study, acquired thermotolerance approach was used, in which seedling were acclimatized first by exposing to moderately elevated temperature, and then leaving them for recovery at optimum temperature before exposing to high temperature (Mueller et al., 2015). Schematic representation of the experimental conditions used in this experiment is shown in **Figure 1A**. Water bath was used as medium for heat stress since water is the better heat conductor. Three independent experiments were performed to assess the seedling viability of *Col-0* and *AtPLC5 OE_2_, OE_3_* and one knockdown (*K_d_)* line of *Arabidopsis* after heat shock at condition referred above. The phenotype of the representative genotypes is presented in **Figure 1B**, while the viability as “greenness” is represented by **Figure 1C**.

The results revealed that all four genotypes survived (100% green seedlings) under control conditions when applied pre-treatment of 37 °C for 60 min and did not show any lethal effects on seedling viability. Subsequently, exposure to heat stress at 45 °C for 150 min was observed to significantly reduce seedling viability compared to control condition. This experiment demonstrated that *AtPLC5* mutants (*OE_2_, OE_3_, K_d_*) exhibited a significant rate of survival upon exposure to heat shock for acquired thermotolerance as compared to wild-type *Col-0*. *AtPLC5* mutants *OE_2_, OE_3_* and *K_d_* indicated around 65-70% survival rate, while for wild-type it was approximated to 40% which means an improvement of around 25-30% in viability for *OE_2_* and *K_d_* mutants of *AtPLC5* seedlings and around 20% for *OE_3_* seedlings. However, the seedlings of *AtPLC5 OE_2_* and *OE_3_* mutants recovered at a better rate, having bigger and greener leaves than the *K_d_* mutant which showed a relatively smaller and pale yellowish green phenotype.

It is assumed that *AtPLC5* over-expression might be involved in acquired thermotolerance as it speeds up the recovery period while knocking down this gene might compromise the rate of seedling recovery and duration of seedling revival.

### 3.3 Chlorophyll contents

**Figure 2:**
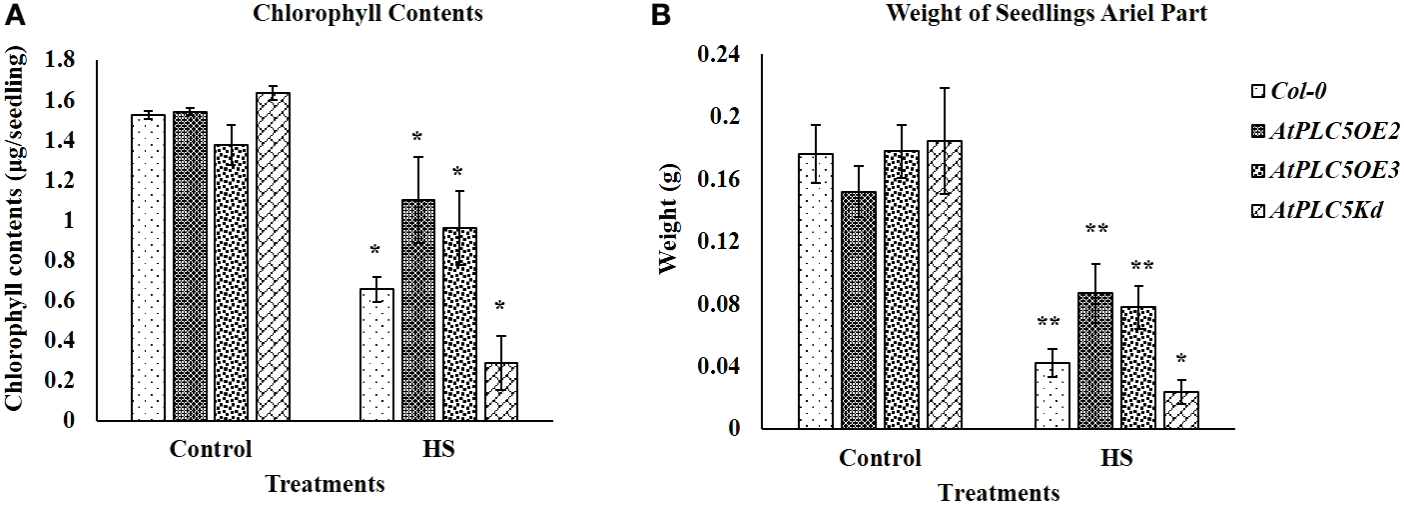
Fifteen days after acquired thermotolerance treatment **(A)** chlorophyll contents (μg/seedling) and **(B)** weight (g) of the aerial part of seedlings were determined. Mean derived from 4 replicates, containing 34 seedlings for each genotype tested. Error bar represent standard deviation of the mean. Students *t-test* (*P < 0.05, **P < 0.01) was used to test the significance.

Chlorophyll contents of seedlings have been considered an additional measure to verify the stay-green character of selected plant phenotype. The results obtained (**Figure 2**) illustrated the same trend which was observed in **Figure 1B**, with the *AtPLC5 OE_2_* mutants, which indicated fast recovery and higher chlorophyll content (**Figure 2A**), seedling weight (ariel part) (**Figure 2B**) and superior plant morphology to other genotypes after heat stress treatment. However, at control conditions, all genotypes show approximately the same level of chlorophyll contents and their ariel part weight.

### 3.4 Root length assay

**Figure 3:**
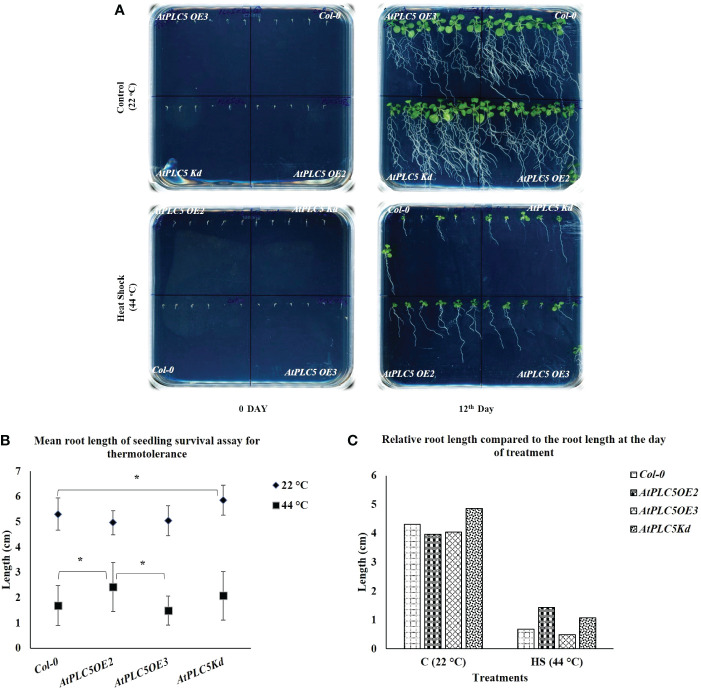
Root growth Assay. Square petri plate divided into 4 parts equally. Six seeds of *Col-0* and *AtPLC5 (OE2, OE3, Kd)* mutants placed on each part with an equal distance. Stratification was done for 2 days in cold dark room at 4 °C, then move to 22 °C (16 h L/8 h D) for growth. Three days old seedlings were treated with 0 min (Control) and 36 min at 44 °C (HS) in a water bath. **(A)** Plates were photographed after 12 days of treatment. **(B)** Mean was derived from 5 replicates, for each genotype tested containing 6 seedlings each. **(C)** Relative root length was also determined at the same time. Student's *t*-test (*P < 0.05) was used to test the level of significance.

Normally, round plates are used for seedling survival assays to be grown horizontally. However, it was almost impossible to explain the behavior of root growth upon heat stress. To tackle this situation, a new experimental setup was used by utilizing the square petri plates placed tilted at an angle of 70° to facilitate the visibility of root growth (**Figure 3A**). For this experiment, three-day old seedlings were treated with 0 min for control and 36 min at 44 °C (Heat Shock), in a water bath. After 12 days, it was observed that at the controlled conditions, *Col-0* and all *AtPLC5* mutants showed highly developed root architecture that was characterized by long main roots from which several lateral roots emerged. After 12 days heat treated seedlings were compared, where all genotypes showed significant decrease in their root development (**Figure 3C**) and *Col-0* was hardly able to develop main and lateral roots as compared to *PLC5 OE_2_, OE_3_, K_d_* lines. Among *AtPLC5* over-expressing lines, *OE_2_* showed longer primary root length with few lateral roots (**Supplementary Figure S1**) as compared to *OE_3_* and *K_d_* mutants (**Figure 3A**).

Smart root plugin was used to measure the main root length and results indicated that there were no significant differences between *Col-0* and all *AtPLC5* over-expressing lines under normal growth conditions. However, significant differences were observed in the average root length of *Col-0* and *AtPLC5* knockdown mutant at controlled conditions (grown at 22 °C) until 12 days (**Figure 3B**). Significant differences were observed in the mean root length of *Col-0* and *AtPLC5 OE_2_* line after 12 days of heat shock (44 °C) exposure, non-significant differences were observed in root length between *Col-0*, *OE_3_* and *K_d_* mutants of *AtPLC5* after 12^th^ day of heat treatment. There was a significant difference between *OE_2_* and *OE_3_* of *PLC5* lines in term of root length of *Arabidopsis thaliana* after 12 days of heat treatment.

### 3.5 Heat induced PPI and PA responses

**Figure 4:**
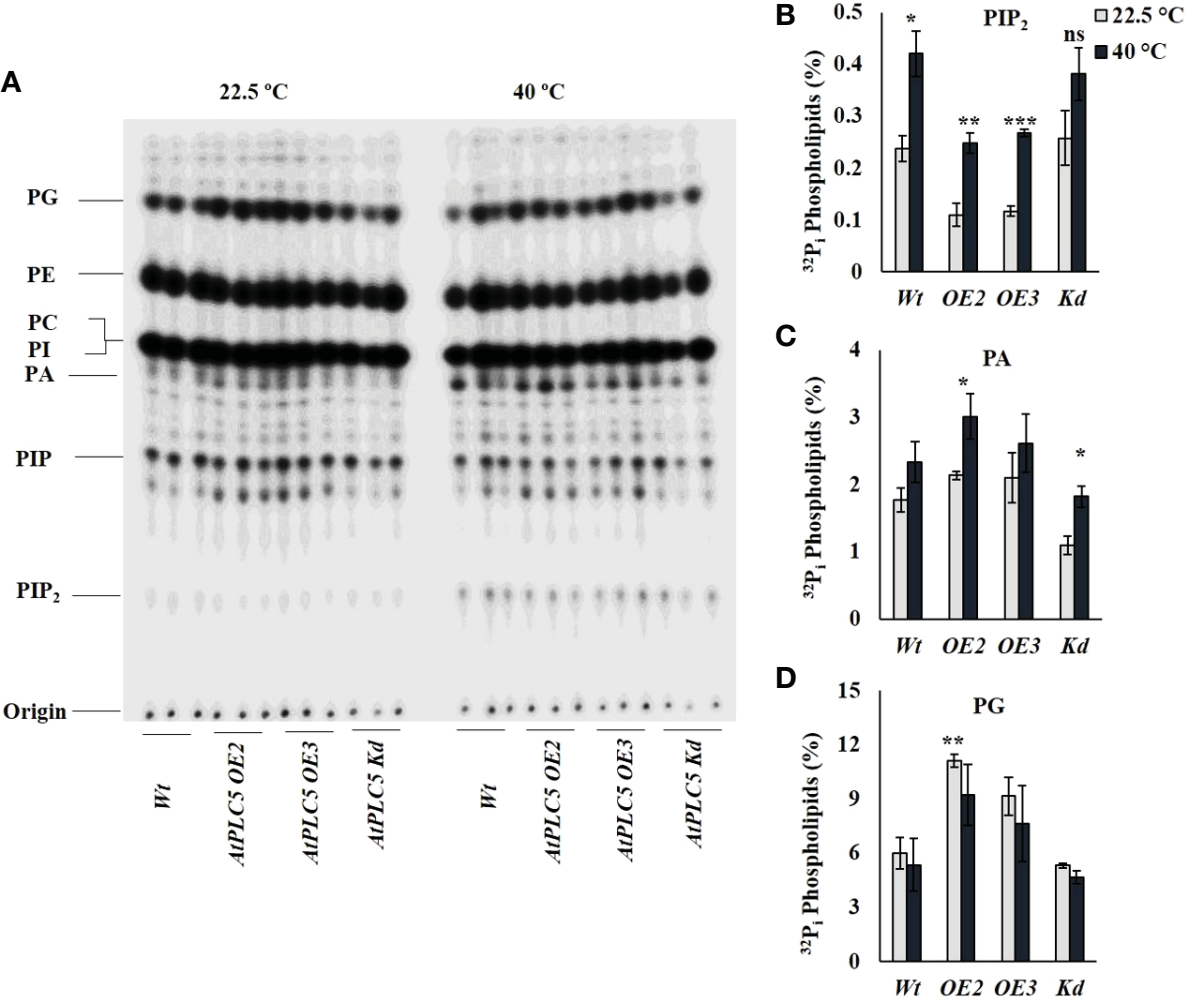
^32^P_i_ lipid profiling of *PLC5* mutants under heat stress. Five days old seedlings were radioactively labeled O/N and treated for 30 min at high temperature (40 °C) using heat block. Lipids were then extracted, analyzed and quantified by phosphoimaging. **(A)** Typical TLC profile of *PLC5* mutants containing lipid extract of 3 seedlings each. **(B)** PIP_2_ **(C)** PA **(D)** PG lipids behavior of *Wt* and *PLC5* mutants under control and heat stressed condition. Data shown are the mean ± SE (n=3). Student's *t*-test (***P < 0.001) was used to test the level of significance.

^32^P_i_ labeling experiment was performed to investigate probable changes in PPI/PA levels as a consequence *PLC5* over-expression and in knockdown *Arabidopsis thaliana* lines when given heat shock (40 °C) for 30 min (**Figure 4A**). Heat treatment to *PLC5 OE* lines elicited a significant increase of ~2.3-fold in PIP_2_ and ~1.4-fold in PA levels as compared to lines grown under control (22.5 °C) conditions (**Figures 4B, C**), validating a constitutively higher PLC activity *in vivo* setting. Moreover, it was witnessed that the absolute level of PIP_2_ in *PLC OE* lines remained below wild-type under controlled conditions. Although a significant increase of ~1.6-fold in PA level was observed in *PLC5 K_d_* mutant upon heat treatment, the absolute level remained below *Wt* and *PLC5 OE* lines. Interestingly, a significant increase of ~1.85-fold was observed in structural lipid PG level (**Figure 4D**) in *PLC5* over-expressed lines as compared to *Wt* under controlled (22.5 °C) conditions. Although the level of PG was decreased slightly due to heat stress the absolute level remains higher than *Wt* and knockdown mutant. This presumably indicating their efficient photosynthetic machinery, as Kobayashi et al., 2015 also reported that the deficiency of PG yielded pale-yellow green phenotype. While the level of PIs remained same under control and heat stressed condition in both *Wt* and *PLC5* mutants.

## 4 Discussion

The extent and rate of global climate change increasingly poses a high-temperature threat to plant survival. Being the primary victim of external stimuli, plasma membrane (PM) guards the plant cells from environmental extremities through downstream signal transduction to generate suitable cellular responses. Membrane constitution and membrane protein operations are all regulated by lipid composition (Rawat et al., 2021). Regulation of phospholipases (PLCs) is not simple; it involves Ca^+2^ signaling, activation of G-proteins and modifications at post-translational stage (Munnik, 2014). PLCs have been investigated extensively for their regulatory role in various biotic and abiotic stress management (Gao et al., 2014). PI(4,5)P which is a presumable substrate of PLC could hardly be detected in flowering plants. However, the rapid elevation in PIP_2_ and PA levels when exposed to heat stress suggest that these lipids are closely associated with thermosensing (Mishkind et al., 2009; Hayes et al., 2021). But still it is unclear how these lipid modifying enzymes are activated by elevated temperature. In this study *PLC5*, one of the nine isoforms of PLC family of *Arabidopsis thaliana*, was tested for thermotolerance to understand its role in heat signaling.

In the present study, phenotypic analysis showed that seedlings of *PLC5 OE* lines exhibited quick and better recovery from heat shock (**Figure 1A**) compared to wild-type and *PLC5 K_d_* seedlings. The phenotype of *PLC5 OE* lines was observed to have bigger and greener leaves as compared to knockdown mutant and wild-type that exhibited smaller leaves of pale-yellow green in color after heat shock, indicating slow or compromised recovery. Recently transgenic wheat containing *AtPLC5* transgene was also reported to retain their stay green character under heat stress (40°C) as compared to *Wt* wheat (Annum et al., 2022). Together these results suggested an involvement of *PLC5* gene in seedling survival under acquired thermotolerance.

Chlorophyll plays a significant role in light harvest, separation of charge and transport in photosynthesis (Kato et al., 2020). The loss of chlorophyll caused by high temperature is associated with heat sensitivity in plants (Shirdelmoghanloo et al., 2016; Zhou et al., 2017). In the present study although a significant decrease in chlorophyll level was observed in *Wt* and *PLC5* mutants when subjected to heat stress, *PLC5 OE* lines showed significantly higher chlorophyll contents as compared to *Wt* and *PLC5* knockdown mutant when exposed to heat stress, indicating higher photosynthetic activity and high temperature tolerance. Whereas, the severe and significant loss of chlorophyll in knockdown mutant of *PLC5* under high temperature showed impaired photosynthetic machinery and temperature sensitivity. Consequently, the chlorophyll contents of wild-type (*Col-0*) and *PLC5* over-expressed and knockdown mutant support the phenotypic observation with significant differences.

Previously, it was reported that abiotic stresses could hinder root development (Zhang et al., 2018b), and similar root growth retardation was observed in case of *PLC5 OE* lines. Under controlled condition (22 °C), *PLC5 K_d_* mutant showed significantly well-developed root architecture with primary and secondary root growth as compared to wild-type (**Figure 3A**), whereas *PLC5 OE* lines revealed reduction in well-developed root architecture which might be the result of decreased PIP_2_ level as it was previously reported that induced *PIP5K3* over-expression in the *PLC5* background rescued the PIP_2_ production and restored root hair phenotype (Van Leeuwen et al., 2007; Zhang et al., 2018b). Similarly, the root growth of *Col-0* and *PLC5* mutants after being subjected to heat shock (44 °C for 36 min) showed a clear reduction in root structure as compared to control (22 °C) condition, still *PLC5* over-expressing lines developed longer main root with fewer secondary roots as compared to *K_d_* mutant that did not develop secondary roots, whereas *Col-0* was found to be hardly successful in developing main root.

Cell membrane, as a permeable barrier, is mainly composed of lipids and proteins. These lipids can be further divided structurally into 3 distinct groups *i.e.*, glycerolipids, sphingolipids and sterol. Among different glycerolipids, phospholipids include Phosphatidylinositol 4,5-bisphosphate (PIP_2_), Phosphatidylinositol monophosphate (PIP), Phosphatidic acid (PA), Phosphatidylinositol (PI), Phosphatidylcholine (PC), Phosphatidylethanolamine (PE), Phosphatidylglycerol (PG), and Cardiolipin (CL) (Abdelrahman et al., 2020). The activation of phospholipid based signaling pathway had been reported to be involved in calcium based heat stress responses (Tuteja and Mahajan, 2007). Although PLC and the subsequent liberation of IP3 upon heat stress could provide mechanism for calcium release, still the signaling of PIP_2_ and PLC is different in animals and plants (Mishkind et al., 2009).

Although PIP_2_ is a genuine animal PLC substrate, it is present in relatively lower concentration in plants and is rarely detected in plasma membranes where most PLC activity is known to be perceived (Munnik, 2014). Whereas, PI4P is abundantly present in plasma membrane suggesting its role as a plant PLC substrate (Vermeer et al., 2017). Obviously, this might be changed under stress conditions where PIP_2_ level is reported to be increased under osmotic stress, salinity stress, and heat stress (Darwish et al., 2009; Zhang et al., 2018b; Annum et al., 2022). In the current study, a clear reduction in PIP_2_ level in *PLC5 OE* lines under normal condition was observed whereas, the level of PA was increased as compared to *Wt* and *PLC5* knockdown mutant which are also in agreement with (Zhang et al., 2018b). Subsequently, a significant increase in PIP_2_ and PA level, when given heat shock (40 °C) has been observed in wild-type and *PLC5* mutants. However, PA level displayed a non-significant increase in *Wt* whereas, a significant increase in *PLC5 OE_2_* which was assumed to an increase in PLC activity. Although a significant increase in PA level was also observed in *PLC5 K_d_* mutant, the total level was less than *Wt* and *PLC5 OE* lines, which might be due to PLD activity.

In plants, PG (phosphatidylglycerol) is mainly present in the thylakoid membrane of chloroplast and plays an important role in photosynthetic electron transport chain (Hagio et al., 2002; Kobayashi et al., 2017). Previous reports suggested the prerequisite activity of PG for chloroplast biogenesis, as its deficiency yielded a pale-yellow green phenotype and became unsuccessful in establishing thylakoid membrane networks inside leaf chloroplast (Haselier et al., 2010; Kobayashi et al., 2015). Interestingly, in the present study, over-expression of *PLC5* resulted in a significant increase in PG level (~1.85-fold) under controlled conditions as compared to *Wt* and knockdown mutant. Although a non-significant decrease was observed in PG level when exposed to heat stress the overall level remains higher, supporting the phenotypic greener color seedling leaves as compared to *PLC5 K_d_* mutant which exhibited pale yellow green phenotype that might be a result of compromised chloroplast biogenesis. Further research in this area is required to identify the exact role of PG against abiotic stresses. And how it contributes in plant phenotypes under abiotic stresses? And to decipher the exact role of PLCs and downstream targets of IPPs, PPIs and PA. Whether PIP_2_ or PIP is an assumed substrate of PLC or PIPK? This work is in the process of extending the current understanding of plant signaling to thermal stress and provides a new perspective on the role of phosphoinositide as an important messenger in this process.

## 5 Conclusion

The seedling survival assay of *PLC5* over-expressing lines of *Arabidopsis thaliana* appeared to be more thermotolerant than *PLC5* knockdown mutant and *Col-0*, indicating the involvement of *PLC5* gene in high temperature tolerance. The chlorophyll analysis and radioactive lipid profiling further support the results of this study, revealing that phosphatidylglycerol (PG) activity which is essential for chloroplast biogenesis and involved in photosynthetic electron transport chain, is increased by ~1.8-fold in *PLC5* over-expressed lines of *Arabidopsis thaliana.* Thus, we assume over-expression of *PLC5* increased thermotolerance and this is caused by the increased hydrolysis of PIP_2_ and highly efficient photosynthetic machinery.

## Data availability statement

The original contributions presented in the study are included in the article/**Supplementary Material**. Further inquiries can be directed to the corresponding author.

## Author contributions

NA was responsible for Methodology, Investigation, Formal analysis, Writing – Original draft preparation, MA validated the data, review and edit the manuscript, MT and ZM critically reviewed the final manuscript and incorporated valuable intellectual input, NAS conceptualized, supervised and approved the final manuscript. All authors contributed to the article and approved the submitted version.
