# Supplementary figures and images for: Physiological responses induced by phospholipase C isoform 5 upon heat stress in Arabidopsis thaliana
Source: Front Plant Sci. 2023 Jan 25;14:1076331. doi: 10.3389/fpls.2023.1076331 (PMC9905699; doi:10.3389/fpls.2023.1076331)

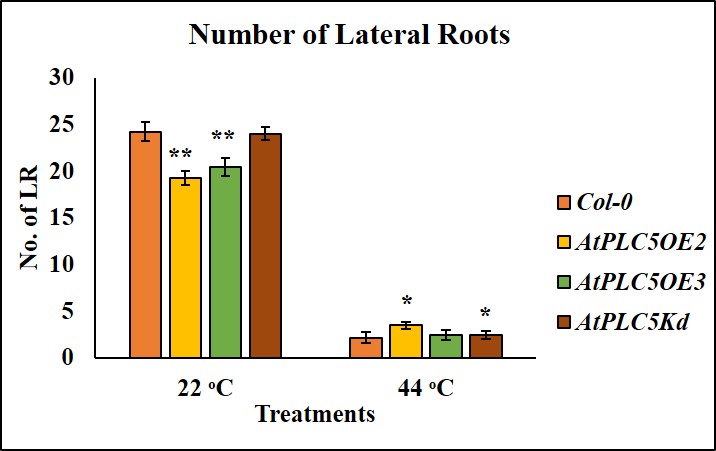

Supplement: Supplementary file 1 [file Image_1.jpeg]

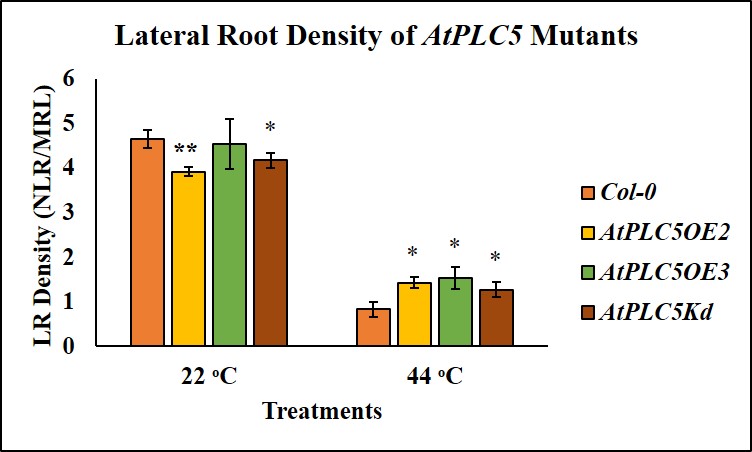

Supplement: Supplementary file 2 [file Image_2.jpeg]
